# Gastrointestinal Tolerance, Growth and Safety of a Partly Fermented Formula with Specific Prebiotics in Healthy Infants: A Double-Blind, Randomized, Controlled Trial

**DOI:** 10.3390/nu11071530

**Published:** 2019-07-05

**Authors:** Alfonso Rodriguez-Herrera, Kelly Mulder, Hetty Bouritius, Rocio Rubio, Antonio Muñoz, Massimo Agosti, Gianluca Lista, Luigi Corvaglia, Thomas Ludwig, Marieke Abrahamse-Berkeveld, Juan L. Perez-Navero

**Affiliations:** 1 Instituto Hispalense de Pediatria, 41013 Sevilla, Spain; 2 Danone Nutricia Research, 3584 CT Utrecht, The Netherlands; 3 Servicio de Pediatría, Hospital Quiron, 08023 Barcelona, Spain; 4 Department of Pediatrics, Hospital Clínico Universitario San Cecilio, 18016 Granada, Spain; 5 Neonatologia e Terapia Intensiva Neonatale, Ospedale Filippo Del Ponte di Varese, 21100 Varese, Italy; 6 Terapia Intensiva Neonatale, Ospedale dei Bambini Vittore Buzzi, ASST-FBF-Sacco, 20154 Milano, Italy; 7 Intensive Therapy Unit, Hospital S. Orsola Malpighi, 40138 Bologna, Italy; 8 Danone Nutricia Research, Singapore 138671, Singapore; 9 Pediatrics Department, Reina Sofia University Hospital, Maimonides Institute for Biomedical research, CIBERER, 14004 Cordoba, Spain

**Keywords:** fermented formula, scGOS/lcFOS, stool characteristics, gastrointestinal symptoms, growth adequacy, crying, sleeping

## Abstract

This study evaluated the effect of a partly fermented infant formula (using the bacterial strains Bifidobacterium breve C50 and Streptococcus thermophilus 065) with a specific prebiotic mixture (short-chain galacto-oligosaccharides (scGOS) and long-chain fructo-oligosaccharides (lcFOS; 9:1)) on the incidence of gastrointestinal symptoms, stool characteristics, sleeping and crying behaviour, growth adequacy and safety. Two-hundred infants ≤28 days of age were assigned either to experimental infant formula containing 30% fermented formula and 0.8 g/100 mL scGOS/lcFOS or to non-fermented control infant formula without scGOS/lcFOS. A group of breastfed infants served as a reference. No relevant differences in parent-reported gastrointestinal symptoms were observed. Stool consistency was softer in the experimental versus control group with values closer to the breastfed reference group. Daily weight gain was equivalent for both formula groups (0.5 SD margins) with growth outcomes close to breastfed infants. No clinically relevant differences in adverse events were observed, apart from a lower investigator-reported prevalence of infantile colic in the experimental versus control group (1.1% vs. 8.7%; *p* < 0.02). Both study formulae are well-tolerated, support an adequate infant growth and are safe for use in healthy term infants. Compared to the control formula, the partly fermented formula with prebiotics induces stool consistencies closer to breastfed infants.

## 1. Introduction

Breastfeeding is the preferred source of nutrition for infants and has been proven to provide a range of short-term and long-term benefits for the child’s nervous, immune, metabolic and gastrointestinal system [1]. Whenever breastfeeding is not possible or ceased, breast milk substitutes should aim to provide nutritional and functional properties as close as possible to those of human milk [2].

Over the past decades, several studies have indicated that (partly) fermented infant milk formulae beneficially impact gastrointestinal function [3,4,5,6,7]. Fermentation processes using food-grade microorganisms generate bioactive compounds, which are also known as postbiotics (adapted from [7,8]). Specific postbiotics are reported to have antimicrobial, antioxidative and immunomodulatory properties [8,9]. Fermented infant formulae using the bacterial strains Bifidobacterium breve C50 (BbC50) and Streptococcus thermophilus 065 (St065) during processing have shown immune and gut health benefits in several clinical studies [4,5,6,10,11]. In parallel, the presence of a specific prebiotic mixture of short-chain galacto-oligosaccharides (scGOS) and long-chain fructo-oligosaccharides (lcFOS; 9:1) in (non-fermented) infant formula was proven to have beneficial effects on stool consistency and frequency, gut microbiota and immune function [12,13,14,15,16]. Previously, combining this specific prebiotic mixture with different dosages of fermented infant formula (using the bacterial strains BbC50 and St065) did not raise any safety concerns in healthy, term infants [17].

The objective of the current randomized, controlled, double-blind, explorative trial was to evaluate the effect of an infant formula combining the specific prebiotic mixture scGOS/lcFOS (9:1; 0.8 g/L) and fermented infant formula (30% of total composition; using BbC50 and St065 during fermentation) on the incidence of gastrointestinal (GI) symptoms, stool characteristics, infant crying and sleeping behaviour as well as on infant growth adequacy and safety. As a reference, a group of fully breastfed infants was included.

## 2. Materials and Methods

### 2.1. Participants

Parents and their infants were recruited from paediatric medical clinics in Italy (3 sites) and Spain (6 sites) from June 2012 to December 2013. Only parents who autonomously decided to exclusively formula feed or with the intention to continue exclusive breastfeeding their infant were informed of the study. Eligible infants were term-born (≥37 and ≤42 weeks gestational age), of normal birth weight (10th to 90th percentile according to applicable growth charts), ≤28 days of age and having a head circumference within +/−2 SD according to the World Health Organization (WHO) growth standards. Infants with a known increased risk of cows’ milk allergy, soy allergy, lactose intolerance, any medical condition that could interfere with study outcomes or having a mother suffering from (gestational) diabetes were excluded from participation. Infants meeting all criteria but fed with an infant formula (IF) containing probiotics or synbiotics prior to study entry were also excluded from participation. Written informed consent was obtained from all parent(s) or guardian(s) before enrolment in the study.

### 2.2. Trial Design

This study was a multi-centre, prospective, double-blind, randomised control trial designed to explore the incidence of GI symptoms, stool characteristics, growth adequacy and safety in healthy, term-born infants up to 17 weeks of age.

Before discharge, after delivery or during a subsequent postnatal hospital visit, as part of the standard care practices in Italy and Spain, parents who autonomously decided to exclusively formula feed or who had the intention to continue exclusive breastfeeding were informed about the current study. Upon enrolment, exclusively IF fed infants were assigned to one of two formulae using a computer-generated randomisation number with country, centre and sex as strata. Formulae were coded by the sponsor (only known to the clinical study supplies manager) using the letter codes A, B, C and D; both the investigators and the infants’ parents were blinded to the formulae and the randomisation details. Inclusion of twins was allowed and were to be randomized to the same product group. As a reference, a group (*n* = 100) of exclusively breastfed infants were to be enrolled in the study. An interactive web-response system was used by the investigator to provide each subject, formula-fed and breastfed infants, with their unique study number when enrolled.

This study was conducted according to the guidelines laid down in the Declaration of Helsinki and all procedures were reviewed and approved by the relevant Ethical Committees in participating countries, for example, the Azienda Ospedaliero-Universitaria di Bolognia for the study site in Bologna. The study was registered with the Dutch Trial Register (wwww.trialregister.nl; NL3308). The study was funded by Danone Nutricia Research, who was also involved in the design and evaluation of the study. An interim analysis was performed to evaluate tolerance, growth and safety outcomes. The independent Data Monitoring Committee concluded that the study could continue as described in the pre-defined protocol.

### 2.3. Study Product

The intervention formulae were comparable in nutritional composition; cow’s milk based, iso-caloric (66 kcal/100 mL) products containing similar amounts of protein (1.2 g/100 mL), lipids (3.4 g/100 mL), vitamins and minerals, manufactured per good manufacturing practices (ISO 22000) and compliant with Directive 2006/141/EC. The experimental IF contained the specific prebiotic mixture scGOS/lcFOS (9:1) and 30% of the total composition was fermented formula. The fermented formula fraction was obtained from a unique fermentation process (Lactofidus^TM^) with two bacterial strains *Bifidobacterium breve* C50 and *Streptococcus thermophilus* 065 generating bioactive compounds; one of these being 3′galactosyllactoses (3′-GL)—an oligosaccharide found in human milk—at a level of ~25 mg/100 mL. The control IF did not contain prebiotics and no fermentation process was applied. Both products had a similar taste, smell and appearance and were manufactured by Danone Nutricia Research (The Netherlands).

### 2.4. Measurements

Since this study was designed as an exploratory trial, no primary parameter was defined. The exploratory outcomes included gastrointestinal symptoms as well as measures of infant growth, stool characteristics, sleeping and crying behaviour, formula intake and adverse events (AEs).

The baseline visit occurred ≤28 days of age and infants were assessed at 4, 8, 13 and 17 weeks of age thereafter. Demographic information and infant characteristics were collected by interview at the baseline visit. Baseline data were collected for stool consistency and frequency, severity of GI symptoms and infant crying duration and frequency by parental recall over the past week. 

At each study visit, infant anthropometrics were measured; the weight for each infant was registered by weighing them naked, on calibrated electronic scales, supine length of infants was registered by using a standard measuring board and a non-stretchable slotted insertion tape was used to measure head circumference. AEs and the use of concomitant medication, drinks and food were documented by the investigators at each visit. For AEs the start and stop date, severity and taken actions were documented. Moreover, the investigators documented the probability of any relationship with the study product.

Daily dairies were filled in by the parents during the entire study duration (up to 17 weeks of age) and recorded stool frequency and consistency as well as crying and sleeping behaviour. Stool consistency was recorded using a 5-point scale (watery, soft pudding-like, soft formed, dry formed, hard pellets) and crying and sleeping behaviour was recorded using a modified Baby day diary with a 24 h bar to document crying and sleeping episodes [17].

In the 7-d period preceding each visit, parents recorded study formula intake and the occurrence and severity of gastrointestinal symptoms (e.g., regurgitation, flatulence, abdominal distension) based on a 4-point scale (absent/mild/moderate/severe). At each visit, the completion of the diaries was discussed with the parents and verified for its completion and plausibility by the investigator. In addition to the parent’s perceived and recorded GI symptoms, incidences of functional gastrointestinal disorders were also evaluated applying adapted Rome III criteria to the daily diary recordings [18,19]. As such, regurgitation was defined as throwing up without force with a frequency of two or more times per day. The incidence of infantile colic was calculated from the diary data as a dichotomous yes/no, with a value per infant per week, with ‘yes’ defined if the infant had ≥3 h of crying for at least 3 days within a 7-day period. Constipation was defined as two or less defecation per week associated with dry hard pellets. Diarrhoea was defined according to WHO criteria as the passage of three or more watery stools per day. In addition to the study visits, a total of three telephone calls were conducted between assessment visits to discuss parental questions, record any illness or medications and to monitor protocol compliance.

### 2.5. Statistics

Despite the exploratory approach, a sample size calculation was performed based on potential differences in the incidence of GI symptoms between intervention groups. The a priori estimates were an incidence of GI symptoms of 55% [20] assuming a 10% difference in the general incidence of GI symptoms between the experimental and control group (α error: 0.1, β error: 0.2). Assuming a 24% attrition rate, a total of 200 subjects, 100 in each intervention group were required.

For all diary data, a daily average or a daily total was calculated for those parameters where more than one entry per day was possible (e.g., GI symptoms, stool consistency, crying duration). All diary data were assigned to specified windows corresponding to the study visits and/or weeks of age. The derived parameters were only calculated if records included at least 3 days of data per week. The specified windows were 14–42 days of age for visit 2, 43–73 days of age for visit 3, 74–104 days of age for visit 4 and 105–133 days of age for visit 5. For week of age, the diary information was assigned to the period of ±3 days the exact days of age (e.g., 4 weeks = 25–31 days).

For comparison of the intervention groups and breastfed reference group with the WHO Child Growth, an analysis of growth parameter *z*-scores using WHO growth trajectories [21] was performed by using a mixed model with adjustment of baseline *z*-score.

Apart from the growth equivalence analysis, all parameters of the two intervention groups were compared using a two-sample t-test or Wilcoxon rank-sum test for continuous data (WMW) and the chi-square test or Fisher’s exact test for categorical data (FE), as appropriate. Equivalence analyses for weight gain, length gain and head circumference gain were performed using Parametric Curves Mixed model (PC) which describes the development of growth parameters over time by a second order polynomial curve, with the stratification factors as a fixed effect and each subject’s intercept and slope as random effects. Equivalence between intervention groups was demonstrated when the two-sided 90%CI of the difference in means in daily gain laid within the pre-defined −0.5 SD to +0.5 SD equivalence margins. The data analysis was conducted with SAS software (SAS Institute Inc., Cary, NC, version 9.4 for Windows). Unless stated otherwise, the per protocol analysis is presented. In the per protocol analyses, eligibility of data was assessed on visit level. In the per protocol growth outcomes analysis (PP-G), data was included of subjects that met the inclusion criteria, were protocol compliant and had at least one post-baseline visit with anthropometric data collection. In addition, apart from protocol compliance, the per protocol analysis of tolerance and several other outcomes required availability of diary data and is referred to as the per protocol tolerance (PP-T) population.

## 3. Results

### 3.1. Subject Characteristics

**Figure 1:**
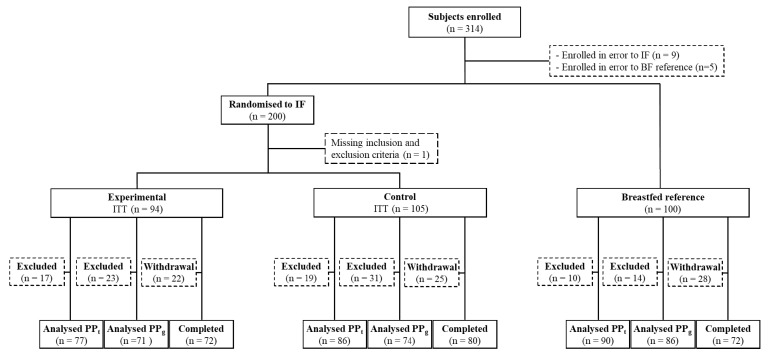
Flow diagram of participants through study, from enrolment to study completion, for Intention to Treat (ITT) and Per Protocol tolerance (PP_t_) and Per Protocol growth (PP_g_) populations. Protocol compliance was based on visit level, explaining the higher number in the PP vs. the Completers population within all three study groups. IF = infant formula; BF = breastfed.

From June 2012 to December 2013, a total of 200 infants were randomized in this trial. A total of 152 infants completed the study of which 72 and 80 infants were part of the experimental and control groups, respectively, resulting in a drop-out rate of 21% (Figure 1). The breastfed reference group included 100 infants of which 72 completed the study.

The number and reasons for early termination were not statistically different between intervention groups and included: no longer wished to participate in the trial (*n* = 44), subjects with an AE (*n* = 14), loss to follow up (*n* = 13), introduced formula in the reference group (*n* = 3) and moved out of the region (*n* = 2). Of the total study population, 5 infants were excluded from all per protocol analyses due to major protocol violations including no study product taken (*n* = 2), cows’ milk allergy (*n* = 1), failure to thrive (*n* = 1) and unknown last intake data (*n* = 1). Three sets of twins were also excluded (*n* = 6) from the per protocol analyses as they were accidentally provided with products with differing study product codes. Additional subjects were excluded from the per protocol population for tolerance outcomes (PP-T) due to lack of any diary data (*n* = 36) and/or introduction of non-study formula (*n* = 8) and from the per protocol population for growth outcomes (PP-G) due to lack of post-baseline visit (*n* = 47), introduction of non-study formula (*n* = 12), not meeting birth weight criteria (*n* = 9), gestational diabetes (*n* = 1), delayed start of study product intake (*n* = 1) or use of glucocorticoids (*n* = 1). Demographic data were not apparently different between the intervention groups for the intention-to-treat population (ITT population; Table 1) as well as both PP populations (data not shown). In addition, infants in the breastfed reference group were slightly older at inclusion in the study (median age 7 days above the intervention groups) and less likely to be born via C-section (Table 1), which is of relevance given its potential impact on GI microbiota.

### 3.2. Study Product Intake

Infants in both intervention groups consumed an increasing amount of formula during the study period. No statistical differences were shown at any timepoint up to 17 weeks of age for volume intake or number of feedings per day between the experimental and control groups in the ITT, PP-G or PP-T populations (data not shown).

### 3.3. Parent-Reported Gastrointestinal Symptoms

**Figure 2:**
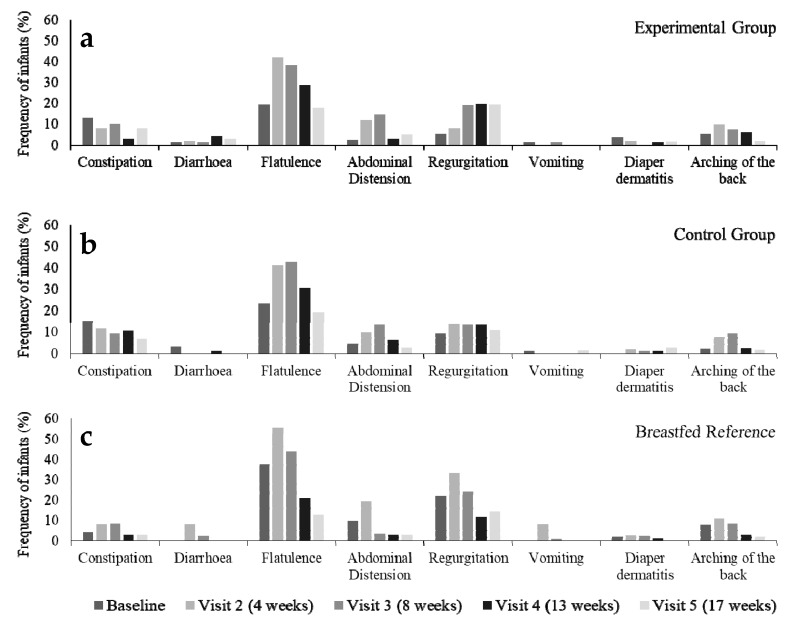
Parent-reported cumulative incidence of GI symptoms with a score of moderate or severe in healthy infants over time for (**a**) the experimental, (**b**) control and (**c**) breastfed reference group in the PP_t_ population. PP_t_ = Per protocol population for diary data on tolerance outcomes.

The overall parent-reported incidence of GI and related symptoms with a score of moderate or severe, postulated to be of clinical relevance, at least once in the study period was not statistically different between intervention groups, with an incidence of 85.7% in the experimental group and 86.0% in the control group. For reference, moderate or severe GI and related symptoms were reported by parents for 84.4% of infants in the breastfed reference group. During the study, the incidence decreased over time for all GI symptoms with a score of moderate or severe in both randomized as well as in the breastfed reference group (Figure 2). Flatulence was the most frequently reported GI symptoms across the study with the highest frequencies at 8 and 13 weeks of age with 30–40% of the infants reported to have moderate to severe flatulence (Figure 2). No statistical differences between the formula groups were observed in the reported cumulative incidences of GI symptoms with a score of moderate or severe for any specific GI symptom during the study (FE *p* > 0.1).

### 3.4. Stool Characteristics

**Figure 3:**
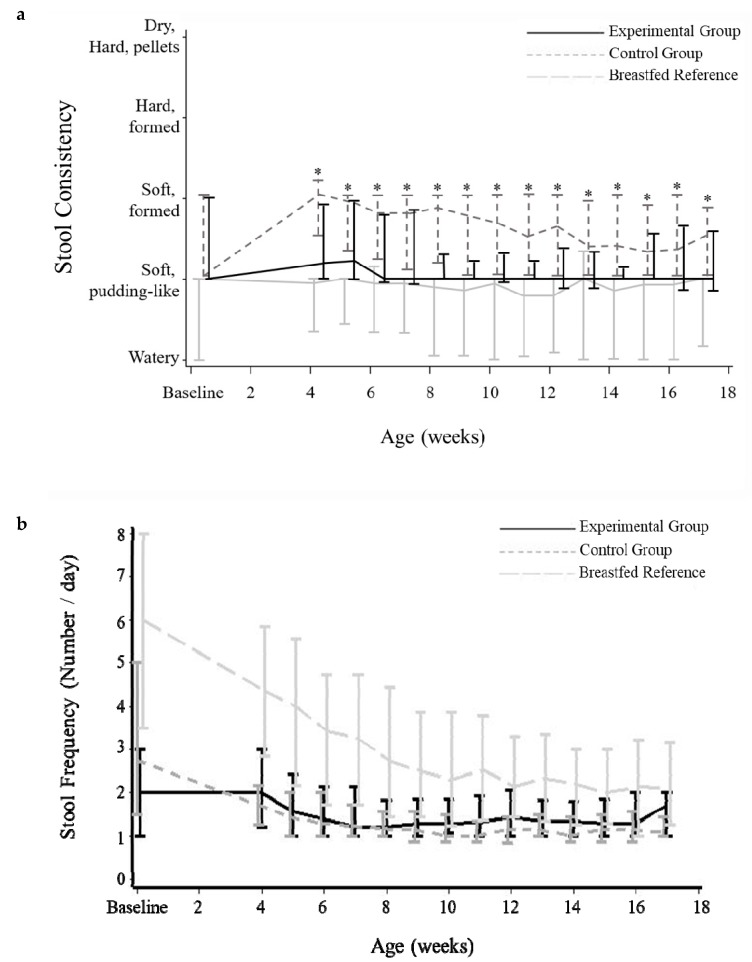
Median stool consistency (**a**) and stool frequency (**b**) for each study group over time in the PP_t_ population. Median (lines) and Q1–Q3 (bars) value are shown per week. Data for baseline were collected as a value per subject as indicated by the parents at the baseline visit. Subsequent values are the mean of each week as reported by parents in the daily diaries. * Statistical difference vs. Experimental Group (*p* < 0.05; Wilcoxon-Mann-Whitney test). PP_t_ = Per protocol population for diary data on tolerance outcomes.

At baseline, stool consistency was not statistically different between the intervention groups (*p* = 0.418), with an overall median (Q1–Q3) of 2.0 (2.0–3.0) and values close to those reported for the breastfed infants (2.0 (1.0–2.0)). After introduction of the intervention formulae, the stool consistency values of infants consuming the experimental formula remained closer to that of the breastfed reference group with consistently and statistically lower values compared to infants in the Control group from 4 weeks of age onwards (WMW *p* < 0.05; Figure 3).

At baseline, no statistical difference in stool frequency was observed between the intervention groups with a median (Q1–Q3) of 2.0 (1.0–3.0) for the experimental group and 2.8 (1.5–5.0) for the control group (WMW *p* = 0.245; Figure 3). Although the median stool frequency in both intervention groups appeared relatively stable over time, values were statistically higher in the experimental versus control group from 9 weeks of age onwards (WMW *p* < 0.05; except at 16 weeks of age). Compared to formula-fed infants, the stool frequency of breastfed infants developed distinctly different over time with a median (Q1–Q3) stool frequency of 6.0 (3.5–8.0) times per day at baseline decreasing over time to a median around 2.0 stools per day at the end of the study.

In line with the reported lower stool consistency values, a slightly higher incidence of diarrhoea, according to WHO criteria, was observed in the experimental versus control group (*p* < 0.05 at 10, 12 and 17 weeks of age) with values from 0 to 8.5% and 0 to 1.4%. However, incidence was higher in breastfed infants with values between 17.0% and 29.9% during the study period. The incidence of constipation according to adapted Rome III criteria was almost nil, with only 2 subjects (1 breastfed, 1 experimental) experiencing one episode throughout the full study period.

### 3.5. Growth Outcomes

**Figure 4:**
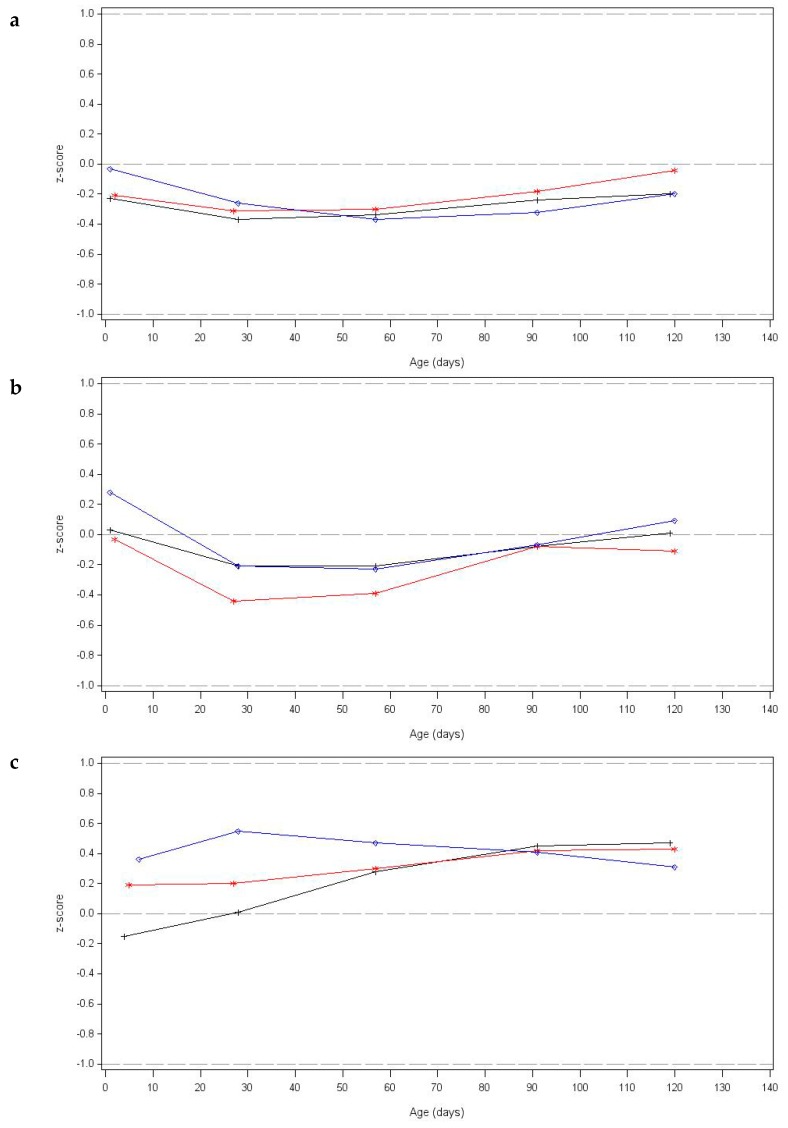
Mean weight-for-age (**a**), length-for-age (**b**) and head circumference-for-age (**c**) World Health Organisation (WHO) growth standard z-scores for the Control (red line), Experimental (blue line) and breastfed reference group (black line).

During the study period, the mean weight-for-age, length-for-age, weight-for-length and head circumference-for-age WHO z-score values of both formula-fed and the breastfed group were close to zero (within a ± 0.5 *z*-score bandwidth; Figure 4), indicative for adequate infant growth.

Equivalence within 0.5 SD boundaries in daily weight gain (g/d) from enrolment to 17 weeks of age was demonstrated for the experimental versus control group (mean (SD) of 28.3 (7.4) g/d versus 30.1 (6.6) g/d) in the PP-G population (difference in estimated means of −1.14 g/d, 90%CI [−2.93, 0.64], SD 6.89) as well as in the ITT population (data not shown). Additionally, no significant differences in body weight were observed between both randomized groups at any of the visits from baseline to 17 weeks of age (Table S1).

Compared to the breastfed reference group (daily weight gain of 27.7 (5.6) g/d) equivalence within 0.5 SD boundaries in daily weight gain (PP-G population) was demonstrated for the experimental group (difference in estimated means of 1.26 g/d, 90% CI [−0.52 to 3.02 g/d], SD 6.39). Daily weight gain was not equivalent in the control versus breastfed reference group of the PP-G population, since the upper confidence limit crossed the upper 0.5 SD equivalence margin (difference in estimated means of 2.43 g/d, 90%CI [0.76 to 4.10 g/d], SD 6.01).

Equivalence in daily length and head circumference gain was demonstrated for both formula groups in the PP and ITT population (data not shown). Additionally, no significant differences in body length or head circumference were observed between both randomized groups at any of the visits from baseline to 17 weeks of age (Table S1). Compared to the breastfed reference group, equivalence in length gain during the study was demonstrated for both formula groups (data not shown). Head circumference gain of both formula groups was not equivalent to the breastfed reference group due to higher gains in the formula groups (mean (SD) of 0.56 (0.13) mm/day and 0.54 (0.12) mm/day for control and experimental groups versus 0.48 (0.12) mm/day for the breastfed reference group).

### 3.6. Adverse Events

A total of 11 serious adverse events (SAEs) were reported for 11 randomized subjects, representing 5.3% and 5.8% of infants in the experimental and control groups, respectively. No statistical differences in percentage of SAEs were observed between formula groups and none of the cases were considered related to the intake of study product. There were no SAEs reported for the breastfed reference group.

Overall, 84 AEs were reported in a total of 62 randomised subjects (31.3%), with the majority classified as mild or moderate. The total number and relatedness of AEs was not statistically different between the formula groups. A total of 27 events in 23 subjects (11.6%) were recorded as (potentially) related to the intervention products, in most cases constipation or colic events. The most commonly observed AEs were gastrointestinal disorders (37 events in 32 subjects) in 13.8%, 18.3% and 6.0% of the subjects in the experimental, control and breastfed reference groups, respectively (Table 2). No statistical differences between formula groups were observed for the total number of gastrointestinal disorders or for individual gastrointestinal disorders. One exception was a statistically lower incidence of investigator reported infantile colic in the experimental group (1.1%) compared to the control group (8.7%; FE *p* = 0.020). For reference, infantile colic was reported for 1.0% of infants in the breastfed reference group.

### 3.7. Infant Crying Frequency and Duration

Daily crying frequency was not statistically different between the formula groups at any time point from baseline to 17 weeks of age and showed a large individual variation evident from the wide ranges reported, that is, ranging from 0 to 17 episodes per day during the first two months after birth (Table S2). In general, the breastfed infants showed quite similar median daily crying frequencies and high individual variations were reported for this group as well.

**Figure 5:**
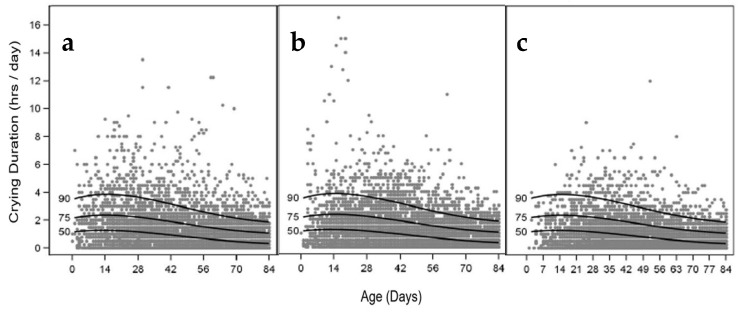
Scatterplots with 90th, 75th and 50th percentile of daily crying duration (hours) up to 12 weeks of age as reported by parents in a daily diary, shown for the (**a**) experimental, (**b**) control and (**c**) breastfed reference groups in the PPt population. PP_t_ = Per protocol population for diary data on tolerance (related) outcomes.

No statistical differences (WMW *p* > 0.05) in total crying duration were demonstrated between formula groups at any time point from baseline to 17 weeks of age (Table S3). The daily crying duration of individual infants was highly variable in the first weeks after birth (Figure 5). Although approximately 90% of the infants were reported to cry less than four hours per day in the first month of life decreasing to two hours per day at 3 months of age, individual reported values ranged from 0 to 16 h per day in that timespan.

In line with the findings on crying, no statistical differences in the incidence of infantile colic based on crying data from the diaries were observed between formula groups during the study period. The incidence of infantile colic showed a consistent decrease from 16–18% at 4 weeks of age to 1–2% at 12 weeks of age and thereafter (data not shown). For comparison, in breastfed infants the incidence of colic based on crying behaviour was on average 6% in the first 8 weeks of life and 1–3% up to 17 weeks of life.

### 3.8. Sleep Frequency and Duration

In general, the median number of parent-reported sleep episodes decreased in all groups over the 17-week study period (Table S4). No statistical differences in the number of reported sleep episodes were observed between the formula groups until 14 weeks of age and a slightly lower median number of sleep episodes was observed in the experimental group (5.5–6.1 n/d) versus control group (6.2–6.7/d) in the weeks thereafter (MWM *p* < 0.07).

Parent-reported sleep duration decreased over the intervention period in all groups, with a range of medians from 13.9–20.0 h/d for the experimental group, 13.9–20.0 h/d for the control group and 13.4–18.5 h/d in the breastfed reference group (Table S5). No relevant differences were observed in sleep duration between the formula groups with values comparable to breastfed infants.

## 4. Discussion

This double-blind, randomized, controlled explorative trial evaluated the effect of a partly fermented infant formula using the bacterial strains *Bifidobacterium breve* C50 and *Streptococcus thermophilus* 065 containing the specific prebiotic mixture scGOS/lcFOS (9:1) on gastrointestinal symptoms, stool characteristics, safety and infant growth and behaviour. Both formulae, experimental and control, were well-tolerated and supported an adequate infant growth. No safety concerns were revealed as reflected by the absence of relevant differences in number, severity, relatedness or type of (S)AEs.

In approximately 85% of the infants, irrespective of infant feeding, at least one occasion of a GI symptom was reported by parents or caregivers as moderate to severe. This is in line with the fact that GI symptoms are known to appear in a significant proportion of infants during the first 6 to 12 months [20,22,23]. As expected, regurgitation, defined as throwing up without force, was one of the most common GI symptoms reported by the parents during this study. The median daily regurgitation frequency was low; with median incidences of once a day or less for formula-fed infants (with no statistical differences between formula groups) and twice a day or less for the breastfed infants (data not shown). The frequency and severity scores of the other GI symptoms were low and, as for regurgitation, its incidence was in line with the findings previously reported for a healthy infant population [20,22,23]. Interestingly, the total incidence of gastrointestinal disorders reported by the investigators as AEs was only 14–18%. In line with previous findings [6], no relevant differences in parent- or investigator-reported incidence or severity of GI symptoms were observed between both formula groups, apart from a statistically lower incidence of infantile colic reported as an adverse event in the experimental group. To conclude, both infant formulae either or not containing fermented formula (and its affiliated postbiotics) and prebiotics are well-tolerated.

In the current study, infant growth outcomes in both formula groups were close to those of breastfed infants of the reference group. Moreover, mean z-scores of all groups were within ±0.5 SD to the WHO growth standards for weight, length and head circumference, indicative for adequate growth. Both formulae were well accepted with no differences in formula consumption between both groups. These findings are in line with previous studies which demonstrated that formulae containing the specific prebiotic mixture and/or fermented formula are well-tolerated and support an adequate infant growth [5,10,17,24]. Noteworthy, the intervention products in the current study had lower protein levels (1.2 g/100 mL) compared to the products used in these previous studies (1.4–1.5 g/100 mL). As indicated by others previously, these protein levels can be sufficient to support adequate infant growth [25], if confirmed by stringent evaluation of safety and suitability [26]. Lastly, none of the SAEs were reported to be related to the study product intake and the percentage of infants with at least one SAE was similar between groups as was the rate and reason for drop-out. Hence, confirming previous findings [17], there are no safety concerns for the use of products combining fermented formula (using Lactofidus^TM^ fermentation process) and a specific prebiotic mixture in healthy, term infants.

The prebiotic mixture scGOS/lcFOS (9:1) present in the experimental formula, has been shown to stimulate the intestinal colonization with bifidobacterial, which results in beneficial effects on immune function and has a stool softening effect [12,13,14,15]. The daily dairies of the current study resulted in >40.000 individual observations for stool characteristics (as well as on crying and sleeping behaviour) of formula and breastfed infant, potentially providing the most extensive reference in the field. As expected, the stool consistency of most of the infants fed the experimental formula in the current study was categorized as ‘soft-pudding-like’ stool (40% to 74%), with a median consistency close to the breastfed infants and lower compared to those fed the control formula. Although a higher incidence of watery stools was reported by parents of the experimental versus control group, values remained below the detected incidences for the breastfed group and were not reflected in any differences in adverse events (diarrhoea, nappy rash) reported by the investigators or reported as diarrhoea in the parental gastrointestinal symptom questionnaires. Based on previous findings [6], the closer to breastfed stool consistency in the experimental group can most likely be largely attributed to the presence of the prebiotic mixture in the formula.

The diary data of the current study confirm the previously reported decrease in infant crying from the second month of life onwards [27]. The peak in total crying duration of 1.3 h per 24 h observed in the study presented here, is in line with the previously reported values of 1.6 h per 24 h [28]. A large variation in crying behaviour was observed in individual infants without any differences in infant crying characteristics between the two randomized groups of the current study. In contrast with both previous findings [6] and the observed differences in investigator reported colic incidences in the current study, the derived incidence of infantile colic based on parent-reported crying duration (defined as ≥3 h/d for ≥3 d/wk) was not different between both randomized groups. The parent-reported crying in the latter as well as the current study was measured as total crying time without separately assessing for unexplainable and inconsolable crying or fussing, which are considered hallmarks of infantile colic. This may explain the differences in colic incidence observed between parent- and investigator reported outcomes. Infantile colic has been associated with alterations in microbiota and low-grade systemic inflammation [29,30,31]. Considering the previously reported immune modulatory properties of both prebiotics and (presumably the postbiotics in) fermented formula [32], their combination is postulated to have a beneficial impact on gut and immune functioning and gastrointestinal symptoms.

## 5. Conclusions

This study evaluated a partly fermented infant milk formula containing the specific prebiotic mixture scGOS/lcFOS (9:1) in healthy term infants. Its findings demonstrate that the formula is well-tolerated, supports an adequate infant growth and is safe for use in healthy term infants. Compared to a control infant formula, the partly fermented formula with prebiotics had stool consistencies closer to those observed in breastfed infants.

## Figures and Tables

**Table 1:** Demographics and baseline characteristics of infants per intervention group of the intention to treat population *.

	Experimental Group	Control Group	Breastfed Reference
	*n* = 94	*n* = 105	*n* = 100
Infant sex, % male/female	50.0/50.0	49.5/50.5	50.0/50.0
Gestational Age (wks), Median (Q1–Q3)	40.0 (38.0–40.0)	39.0 (38.0–40.0)	40.0 (39.0–41.0)
Country, % Italy/Spain	5.3/94.7	7.6/92.4	5.0/95.0
Birth weight (g), Median (Q1–Q3)	3265 (2960–3550)	3170 (2932–3520)	3350 (3015–3650)
Birth length (g), Median (Q1–Q3)	50.0 (48.5–51.0)	50.0 (48.0–51.0)	50.0 (49.5–52.0)
Mode of delivery, % vaginal/c-section ^1^	68.1/31.9	61.0/38.1	82.0/17.0
Order in household, % 1, 2, ≥3	86.2/12.8/1.1	81.9/15.2/2.9	84.0/11.1/5.0
Maternal age (years; mean ± SD)	32.9 ± 5.0	33.2 ± 5.0	32.3 ± 3.8
Maternal BMI (kg/m^2^; mean ± SD)	26.0 ± 5.8	25.2 ± 4.8	24.6 ± 5.0
Maternal education, %			
Primary School	15	18	11
High School	47	51	43
University	36	29	41
Age at inclusion (days), Median (Q1–Q3)	14.0 (5.0–24.0)	15.0 (5.0–25.0)	23.0 (9.5–27.0)

^1^ Data missing for one infant in the control group. * No statistical significant differences (P < 0.05) in characteristics were observed between both intervention groups.

**Table 2:** Gastrointestinal disorders reported by investigators as Adverse Events (AEs) in the study population (AST).

Adverse Events	Experimental Group *n* = 94	Control Group *n* = 104		Breastfed Reference *n* = 105
	*n* (%)	*n* (%)	*p*-Value	*n* (%)
Any Gastrointestinal Event	13 (13.8)	19 (18.3)	0.443	6 (6.0)
Abdominal Pain	1 (1.1)	0 (0)	0.475	0 (0)
Constipation	6 (6.4)	9 (8.7)	0.600	3 (3.0)
Diarrhoea	1 (1.1)	0 (0)	0.475	0 (0)
Flatulence	2 (2.1)	1 (1.0)	0.605	0 (0)
Gastroesophageal Reflux Disease	2 (2.1)	3 (2.9)	1.000	2 (2.0)
Infantile Colic	1 (1.1) *	9 (8.7)	0.020	1 (1.0)
Regurgitation	1 (1.1)	0 (0)	0.475	1 (1.0)

Adverse events are presented here by preferred term. Investigators reported all adverse events based on their own discretion. AST = All subjects treated. * Statistically different from control group (*p* < 0.05 Fisher’s exact test).

## Supplementary Materials

The following are available online at https://www.mdpi.com/2072-6643/11/7/1530/s1, Table S1. Anthropometric outcomes per study group in the per protocol population (mean ± SD), Table S2. Summary of crying frequency per week of age (episodes/day), Table S3. Summary of crying duration per week of age (hours/day), Table S4. Summary of sleeping frequency per week of age (episodes/day), Table S5. Summary of sleeping duration per week of age (hours/day).
