# Supplementary material for: Gastrointestinal Tolerance, Growth and Safety of a Partly Fermented Formula with Specific Prebiotics in Healthy Infants: A Double-Blind, Randomized, Controlled Trial
Source: Nutrients. 2019 Jul 5;11(7):1530. doi: 10.3390/nu11071530 (PMC6683277; doi:10.3390/nu11071530)
Supplement: Supplementary file 1 [file nutrients-11-01530-s001.pdf]

Table 1. Anthropometric outcomes per study group in the per protocol population (mean ± SD).

	Experimental Group [N = 71]	Control Group [N = 74]	Breastfed reference [N = 86]
Body weight (g)			
Baseline	3619 ± 587	3561 ± 540	3810 ± 526
4 wks	4090 ± 422	4147 ± 495	4082 ± 402
8 wks	5026 ± 593	5060 ± 561	5030 ± 569
13 wks	5976 ± 689	6006 ± 723	5940 ± 634
17 wks	6544 ± 775	6681 ± 806	6607 ± 651
Body length (cm)			
Baseline	51.7 ± 2.4	51.3 ± 2.1	52.3 ± 2.2
4 wks	53.8 ± 2.0	53.2 ± 1.8	53.9 ± 1.9
8 wks	56.9 ± 2.0	56.7 ± 2.4	57.0 ± 2.2
13 wks	60.5 ± 2.2	60.4 ± 2.0	60.6 ± 2.3
17 wks	62.8 ± 2.1	62.6 ± 2.1	63.2 ± 2.4
Head circumference			
Baseline	35.7 ± 1.7	35.8 ± 1.6	36.7 ± 1.5
4 wks	36.7 ± 1.2	37.0 ± 1.3	37.3 ± 0.9
8 wks	38.8 ± 1.4	38.9 ± 1.3	39.1 ± 1.1
13 wks	40.6 ± 1.5	40.5 ± 1.3	40.6 ± 1.2
17 wks	41.6 ± 1.8	41.6 ± 1.3	41.5 ± 1.3

*P<0.05; significant difference between Experimental and Control group.

Table 2. Summary of crying frequency per week of age (episodes/ day)[1].

Age		Experimental Group<br>N = 77	Control Group<br>N = 86	Breastfed reference<br>N = 90
Baseline	Min-Max (n)	0.0 - 10.0 (69)	0.0 - 12.0 (83)	0.0 - 24.0 (84)
	Median (Q1-Q3)	6.0 (3.0 - 8.0)	5.0 (1.0 - 8.0)	5.0 (2.0 - 8.0)
4 wks	Min-Max (n)	0.0 - 11.5 (55)	0.0 - 13.0 (61)	0.0 - 10.8 (68)
	Median (Q1-Q3)	2.5 (1.0 - 4.6)	2.6 (1.0 - 5.2)	2.8 (1.3 - 4.6)
5 wks	Min-Max (n)	0.0 - 14.1 (67)	0.0 - 12.0 (74)	0.0 - 10.4 (84)
	Median (Q1-Q3)	2.7 (1.0 - 4.7)	2.8 (1.0 - 5.4)	2.7 (1.4 - 4.1)
6 wks	Min-Max (n)	0.0 - 15.6 (66)	0.0 - 14.0 (77)	0.0 - 11.0 (84)
	Median (Q1-Q3)	2.5 (1.3 - 4.3)	2.3 (1.1 - 4.4)	2.3 (0.8 - 4.2)
7 wks	Min-Max (n)	0.0 - 17.4 (67)	0.0 - 12.9 (75)	0.0 - 9.2 (80)
	Median (Q1-Q3)	2.4 (0.9 - 4.3)	2.0 (0.6 - 3.7)	1.9 (0.7 - 3.8)
8 wks	Min-Max (n)	0.0 - 15.8 (65)	0.0 - 11.3 (76)	0.0 - 8.7 (78)
	Median (Q1-Q3)	2.0 (0.7 - 4.3)	1.8 (0.7 - 4.1)	1.7 (0.7 - 3.5)
9 wks	Min-Max (n)	0.0 - 11.6 (66)	0.0 - 11.4 (76)	0.0 - 8.6 (73)
	Median (Q1-Q3)	1.9 (0.5 - 3.9)	1.6 (0.4 - 4.6)	1.3 (0.6 - 2.5)
10 Wks	Min-Max (n)	0.0 - 9.1 (64)	0.0 - 9.3 (75)	0.0 - 9.1 (71)
	Median (Q1-Q3)	1.7 (0.6 - 4.1)	1.4 (0.4 - 3.4)	1.3 (0.1 - 2.4)
11 wks	Min-Max (n)	0.0 - 11.3 (64)	0.0 - 9.9 (75)	0.0 - 9.7 (68)
	Median (Q1-Q3)	1.6 (0.4 - 3.7)	0.9 (0.3 - 3.4)	0.9 (0.3 - 2.4)
12 wks	Min-Max (n)	0.0 - 8.9 (64)	0.0 - 9.0 (75)	0.0 - 10.6 (67)
	Median (Q1-Q3)	1.3 (0.4 - 2.9)	1.0 (0.3 - 3.4)	1.0 (0.3 - 2.6)
13 wks	Min-Max (n)	0.0 - 10.7 (63)	0.0 - 10.3 (74)	0.0 - 10.7 (67)
	Median (Q1-Q3)	1.5 (0.5 - 3.1)	0.9 (0.0 - 3.6)	1.1 (0.2 - 2.2)
14 wks	Min-Max (n)	0.0 - 9.0 (64)	0.0 - 8.0 (70)	0.0 - 9.5 (64)
	Median (Q1-Q3)	1.1 (0.3 - 2.8)	0.8 (0.1 - 3.6)	0.8 (0.3 - 2.7)
15 wks	Min-Max (n)	0.0 - 8.4 (63)	0.0 - 7.4 (72)	0.0 - 11.6 (63)
	Median (Q1-Q3)	1.3 (0.3 - 3.0)	0.6 (0.0 - 3.6)	0.9 (0.0 - 2.7)
16 wks	Min-Max (n)	0.0 - 9.6 (62)	0.0 - 7.7 (72)	0.0 - 11.7 (63)
	Median (Q1-Q3)	1.4 (0.4 - 3.8)	0.8 (0.0 - 3.0)	0.9 (0.2 - 2.6)
17 wks	Min-Max (n)	0.0 - 7.3 (29)	0.0 - 6.5 (40)	0.0 - 11.1 (43)
	Median (Q1-Q3)	1.3 (0.2 - 2.6)	0.5 (0.0 - 2.5)	0.7 (0.1 - 2.0)

[1]Based on parent-reported data in the PP_t population (per protocol for diary data on tolerance (related) outcomes). * Statistical difference vs Control $P < 0.05$ ‡Trend for statistical difference vs Control $0.05 < P < 0.10$.

Table 3. Summary of crying duration per week of age (hours/ day) [1.]

Age		Experimental group	Control group	Breastfed reference
		N = 77	N = 86	N = 90
Baseline	Min-Max (n)	0.0 - 6.0 (71)	0.0 - 8.5 (84)	0.0 - 3.0 (87)
	Median (Q1-Q3)	0.7 (0.2 - 1.3)	0.6 (0.2 - 1.8)	0.5 (0.2 - 1.0)
4 wks	Min-Max (n)	0.0 - 7.1 (55)	0.0 - 5.2 (61)	0.0 - 4.3 (68)
	Median (Q1-Q3)	1.2 (0.5 - 2.6)	1.2 (0.5 - 2.1)	1.1 (0.4 - 1.7)
5 wks	Min-Max (n)	0.0 - 5.6 (67)	0.0 - 5.4 (74)	0.0 - 5.1 (84)
	Median (Q1-Q3)	1.3 (0.5 - 2.3)	1.3 (0.5 - 2.2)	0.9 (0.4 - 1.8)
6 wks	Min-Max (n)	0.0 - 6.7 (66)	0.0 - 5.0 (77)	0.0 - 5.6 (84)
	Median (Q1-Q3)	1.0 (0.5 - 2.0)	1.3 (0.5 - 2.1)	0.8 (0.3 - 1.4)
7 wks	Min-Max (n)	0.0 - 5.8 (67)	0.0 - 4.5 (75)	0.0 - 4.7 (80)
	Median (Q1-Q3)	1.0 (0.4 - 1.8)	0.8 (0.3 - 2.0)	0.7 (0.2 - 1.4)
8 wks	Min-Max (n)	0.0 - 7.9 (65)	0.0 - 5.5 (76)	0.0 - 4.4 (78)
	Median (Q1-Q3)	0.8 (0.3 - 1.6)	0.8 (0.3 - 1.7)	0.6 (0.2 - 1.2)
9 wks	Min-Max (n)	0.0 - 5.5 (66)	0.0 - 3.9 (76)	0.0 - 3.1 (73)
	Median (Q1-Q3)	0.8 (0.2 - 1.6)	0.6 (0.2 - 1.6)	0.5 (0.2 - 0.9)
10 wks	Min-Max (n)	0.0 - 2.9 (64)	0.0 - 2.9 (75)	0.0 - 3.4 (71)
	Median (Q1-Q3)	0.7 (0.2 - 1.5)	0.6 (0.2 - 1.3)	0.4 (0.1 - 0.8)
11 wks	Min-Max (n)	0.0 - 3.5 (64)	0.0 - 3.1 (75)	0.0 - 2.8 (68)
	Median (Q1-Q3)	0.5 (0.2 - 1.5)	0.4 (0.1 - 1.1)	0.3 (0.1 - 0.8)
12 wks	Min-Max (n)	0.0 - 2.5 (64)	0.0 - 2.8 (75)	0.0 - 2.8 (67)
	Median (Q1-Q3)	0.7 (0.2 - 1.1)	0.4 (0.1 - 1.2)	0.3 (0.1 - 0.9)
13 wks	Min-Max (n)	0.0 - 2.8 (63)	0.0 - 2.6 (74)	0.0 - 2.9 (67)
	Median (Q1-Q3)	0.5 (0.2 - 1.3)	0.3 (0.0 - 1.1)	0.3 (0.1 - 0.8)
14 wks	Min-Max (n)	0.0 - 2.7 (64)	0.0 - 2.3 (70)	0.0 - 2.5 (64)
	Median (Q1-Q3)	0.4 (0.1 - 1.1)	0.3 (0.0 - 1.1)	0.3 (0.1 - 0.9)
15 wks	Min-Max (n)	0.0 - 2.1 (63)	0.0 - 3.5 (72)	0.0 - 3.7 (63)
	Median (Q1-Q3)	0.5 (0.1 - 1.1)	0.3 (0.0 - 1.0)	0.3 (0.0 - 0.9)
16 wks	Min-Max (n)	0.0 - 2.6 (62)	0.0 - 1.9 (72)	0.0 - 3.2 (63)
	Median (Q1-Q3)	0.6 (0.1 - 1.2)	0.3 (0.0 - 1.2)	0.3 (0.1 - 0.8)
17 wks	Min-Max (n)	0.0 - 2.0 (29)	0.0 - 1.9 (40)	0.0 - 3.0 (43)
	Median (Q1-Q3)	0.5 (0.1 - 0.9)	0.2 (0.0 - 0.8)	0.2 (0.0 - 0.6)

[1]Based on parent-reported data in the PP_t population (per protocol for diary data on tolerance (related) outcomes). * Statistical difference vs Control P < 0.05 ‡Trend for statistical difference vs Control 0.05 < P < 0.10.

Table 4. Summary of sleeping frequency per week of age (episodes/ day) [1].

Age		Experimental Group N = 77	Control Group N = 86	Breastfed reference N = 90
Baseline	Min-Max (n)	0.0 - 10.0 (66)	0.0 - 12.0 (79)	0.0 - 24.0 (81)
	Median (Q1-Q3)	8.0 (5.0 - 8.0)	7.0 (5.0 - 8.0)	8.0 (6.0-8.0)
4 wks	Min-Max (n)	1.8 - 10.3 (55)	2.6 - 10.9 (61)	4.6 - 13.0 (68)
	Median (Q1-Q3)	7.2 (6.3 - 8.5)	7.3 (6.0 - 8.0)	7.5 (6.3 - 8.7)
5 wks	Min-Max (n)	3.4 - 12.3 (67)	3.0 - 11.0 (74)	4.6 - 13.4 (84)
	Median (Q1-Q3)	7.5 (6.3 - 8.1)	7.2 (6.7 - 8.3)	7.6 (6.7 - 8.9)
6 wks	Min-Max (n)	3.4 - 11.4 (66)	2.7 - 10.7 (77)	4.1 - 14.7 (84)
	Median (Q1-Q3)	7.0 (6.0 - 8.1)	7.3 (6.6 - 8.0)	8.0 (6.6 - 9.0)
7 wks	Min-Max (n)	3.8 - 13.0 (67)	2.1 - 10.7 (75)	3.3 - 12.0 (80)
	Median (Q1-Q3)	7.1 (6.1 - 8.3)	7.0 (6.1 - 7.7)	7.6 (6.9 - 9.0)
8 wks	Min-Max (n)	3.8 - 16.0 (65)	2.1 - 9.8 (76)	3.9 - 12.2 (78)
	Median (Q1-Q3)	6.7 (5.3 - 7.8)	6.9 (6.3 - 7.8)	7.7 (6.3 - 9.0)
9 wks	Min-Max (n)	3.7 - 11.3 (66)	2.9 - 10.0 (76)	3.7 - 11.9 (73)
	Median (Q1-Q3)	6.7 (5.6 - 8.2)	7.0 (6.3 - 7.6)	7.6 (6.6 - 8.7)
10 wks	Min-Max (n)	3.9 - 11.7 (64)	2.8 - 9.6 (75)	3.4 - 11.6 (71)
	Median (Q1-Q3)	6.6 (5.5 - 7.9)	6.7 (5.9 - 7.6)	7.7 (6.4 - 8.7)
11 wks	Min-Max (n)	3.4 - 11.1 (64)	3.0 - 9.3 (75)	3.9 - 12.1 (68)
	Median (Q1-Q3)	6.7 (5.5 - 7.6)	6.7 (6.0 - 7.3)	7.4 (6.3 - 8.7)
12 wks	Min-Max (n)	3.9 - 9.6 (64)	3.6 - 8.5 (75)	4.0 - 12.9 (67)
	Median (Q1-Q3)	6.5 (5.6 - 7.6)	6.7 (6.0 - 7.4)	7.3 (6.7 - 8.4)
13 wks	Min-Max (n)	3.0 - 11.2 (63)	3.7 - 10.5 (74)	3.6 - 12.8 (67)
	Median (Q1-Q3)	6.5 (5.4 - 7.5)	6.5 (5.7 - 7.3)	7.5 (6.2 - 8.2)
14 wks	Min-Max (n)	3.0 - 9.1 (64)	3.8 - 8.9 (70)	4.0 - 11.1 (64)
	Median (Q1-Q3)	6.1 (5.1 - 6.9) *	6.7 (5.7 - 7.4)	7.0 (6.0 - 7.9)
15 wks	Min-Max (n)	3.6 - 9.7 (63)	4.0 - 9.1 (72)	3.5 - 10.7 (63)
	Median (Q1-Q3)	6.1 (5.0 - 6.9) ‡	6.4 (5.6 - 7.1)	6.9 (6.0 - 7.9)
16 wks	Min-Max (n)	3.9 - 8.7 (62)	3.4 - 8.8 (72)	3.6 - 11.0 (63)
	Median (Q1-Q3)	5.8 (5.0 - 7.1) ‡	6.4 (5.6 - 7.2)	7.0 (6.0 - 7.9)
17 wks	Min-Max (n)	4.0 - 9.3 (29)	3.0 - 9.0 (40)	3.3 - 11.0 (43)
	Median (Q1-Q3)	5.5 (5.0 - 6.3) *	6.2 (5.7 - 6.8)	6.7 (6.0 - 8.3)

[1]Based on parent-reported data in the PP_t population (per protocol for diary data on tolerance (related) outcomes). * Statistical difference vs Control $P < 0.05$ ‡Trend for statistical difference vs Control $0.05 < P < 0.10$.

Table 5. Summary of sleeping duration per week of age (hours/ day) [1.]

Age		Experimental Group	Control Group	Breastfed reference
		N = 77	N = 86	N = 90
Baseline	Min-Max (n)	3.0 - 23.0 (71)	7.0 - 23.0 (83)	2.0 - 24.0 (85)
	Median(Q1-Q3)	20.0 (17.0 - 21.0)	20.0 (18.0 - 22.0)	18.5 (16.0 - 20.0)
4 wks	Min-Max (n)	10.9 - 22.2 (55)	9.6 - 19.3 (61)	9.3 - 18.2 (68)
	Median(Q1-Q3)	15.0 (12.5 - 16.7)	15.0 (13.5 - 16.3)	14.0 (12.8 - 15.0)
5 wks	Min-Max (n)	10.6 - 21.2 (67)	10.5 - 18.4 (74)	10.6 - 18.4 (84)
	Median(Q1-Q3)	14.4 (12.7 - 15.5) *	15.3 (14.0 - 16.1)	14.2 (12.9 - 15.4)
6 wks	Min-Max (n)	10.2 - 19.8 (66)	10.0 - 19.3 (77)	8.6 - 17.9 (84)
	Median(Q1-Q3)	14.1 (12.8 - 16.0)	14.9 (13.5 - 16.0)	14.3 (12.8 - 15.4)
7 wks	Min-Max (n)	8.7 - 20.5 (67)	10.7 - 19.4 (75)	8.2 - 17.5 (80)
	Median(Q1-Q3)	14.2 (12.6 - 15.9)	14.8 (12.7 - 15.6)	13.8 (12.9 - 15.5)
8 wks	Min-Max (n)	11.7 - 19.4 (65)	9.4 - 19.5 (76)	6.9 - 18.0 (78)
	Median(Q1-Q3)	14.1 (13.1 - 15.9)	14.5 (13.4 - 16.3)	14.2 (13.1 - 15.3)
9 wks	Min-Max (n)	10.6 - 18.0 (66)	11.5 - 18.4 (76)	7.6 - 16.7 (73)
	Median(Q1-Q3)	14.4 (13.2 - 15.6)	14.7 (13.1 - 15.8)	14.1 (13.1 - 15.2)
10 wks	Min-Max (n)	11.0 - 18.9 (64)	10.2 - 18.8 (75)	8.4 - 17.4 (71)
	Median(Q1-Q3)	14.1 (13.0 - 15.5)	14.2 (13.0 - 15.6)	14.1 (12.8 - 14.9)
11 wks	Min-Max (n)	11.4 - 18.7 (64)	10.8 - 19.4 (75)	8.5 - 17.2 (68)
	Median(Q1-Q3)	14.1 (12.8 - 15.6)	14.2 (12.7 - 15.6)	13.9 (12.9 - 15.0)
12 wks	Min-Max (n)	10.5 - 19.0 (64)	11.2 - 19.2 (75)	8.8 - 18.0 (67)
	Median(Q1-Q3)	14.0 (13.1 - 15.3)	14.2 (12.8 - 15.6)	13.8 (13.0 - 15.0)
13 wks	Min-Max (n)	11.5 - 17.1 (63)	10.0 - 18.0 (74)	9.0 - 17.9 (67)
	Median(Q1-Q3)	14.2 (13.2 - 15.3)	14.1 (12.9 - 15.1)	13.7 (12.7 - 15.0)
14 wks	Min-Max (n)	11.1 - 17.3 (64)	8.8 - 17.9 (70)	8.1 - 16.2 (64)
	Median Q1-Q3)	14.2 (13.3 - 15.2)	14.0 (12.9 - 14.5)	13.4 (12.4 - 14.4)
15 wks	Min-Max (n)	12.0 - 17.1 (63)	2.4 - 17.6 (72)	8.3 - 16.7 (63)
	Median(Q1-Q3)	13.7 (12.9 - 14.9)	14.1 (12.9 - 15.0)	13.4 (12.5 - 14.2)
16 wks	Min-Max (n)	11.3 - 16.8 (62)	2.3 - 17.7 (72)	8.3 - 15.6 (63)
	Median(Q1-Q3)	13.8 (13.0 - 14.8)	13.8 (12.7 - 14.8)	13.3 (12.5 - 13.9)
17 wks	Min-Max (n)	11.7 - 16.8 (29)	3.1 - 17.8 (40)	9.6 - 16.4 (43)
	Median(Q1-Q3)	13.9 (13.0 - 15.3)	13.9 (13.2 - 14.7)	13.4 (12.7 - 14.2)

[1]Based on parent-reported data in the PP_t population (per protocol for diary data on tolerance (related) outcomes). * Statistical difference vs Control $P < 0.05$ ‡Trend for statistical difference vs Control $0.05 < P < 0.10$.
